# Revealing Differentially Expressed Genes and Identifying Effector Proteins of Puccinia striiformis f. sp. *tritici* in Response to High-Temperature Seedling Plant Resistance of Wheat Based on Transcriptome Sequencing

**DOI:** 10.1128/mSphere.00096-20

**Published:** 2020-06-24

**Authors:** Fei Tao, Yangshan Hu, Chang Su, Juan Li, Lili Guo, Xiangming Xu, Xianming Chen, Hongsheng Shang, Xiaoping Hu

**Affiliations:** a State Key Laboratory of Crop Stress Biology for Arid Areas and College of Plant Protection, Northwest A&F University, Yangling, Shaanxi, China; b Biocontrol Engineering Laboratory of Crop Diseases and Pests of Gansu Province, College of Plant Protection, Gansu Agricultural University, Lanzhou, Gansu, China; c Pest & Pathogen Ecology, NIAB EMR, East Malling, Kent, United Kingdom; d Agricultural Research Service, United States Department of Agriculture and Department of Plant Pathology, Washington State University, Pullman, Washington, USA; Yonsei University

**Keywords:** *Puccinia striiformis* f. sp. *tritici*, wheat (*Triticum aestivum* L.), high-temperature seedling-plant (HTSP) resistance, transcript profiling, fungal effector protein

## Abstract

Stripe rust, caused by Puccinia striiformis f. sp. *tritici* (*Pst*), is one of the most important diseases of wheat (Triticum aestivum L.) globally. Recently, more aggressive *Pst* races have evolved to acquire new virulence profiles and are adapted better to high temperature than most of the previous races. Breeding cultivars with durable high-temperature seedling-plant (HTSP) resistance is an important strategy for controlling stripe rust. Understanding the mechanism of wheat HTSP resistance against *Pst* is important for more efficient breeding to improve host resistance. In the present study, transcriptomic analysis identified 25 *Pst* differentially expressed genes (DEGs) that were involved in the HTSP resistance in wheat cultivar Xiaoyan6 (XY6). Functional annotation indicated that these DEGs are related to membrane proteins, mRNA binding proteins, cell membrane transporters, and synthesis of cell nitrogen compounds. Among these DEGs, a candidate effector, *PstCEP1* (*PSTG_13342*), was identified and cloned, and its function was verified. Barley stripe mosaic virus (BSMV)-mediated host-induced gene silencing (HIGS) of *PstCEP1* reduced *Pst* virulence. Signal peptide verification and functional testing in Nicotiana benthamiana indicated that PstCEP1 is a secreted protein and has the function of suppressing programmed cell death (PCD). PstCEP1 as a candidate effector was further supported by type three secretion system (TTSS)-mediated overexpression responding to wheat HTSP resistance via affecting the pathogen-associated molecular pattern (PAMP)-triggered immunity (PTI) and effector-triggered immunity (ETI).

**IMPORTANCE** In the present study, we performed transcriptomic analysis to identify differentially expressed genes and effector proteins of Puccinia striiformis f. sp. *tritici* (*Pst*) in response to the high-temperature seedling-plant (HTSP) resistance in wheat. Experimental validation confirmed the function of the highest upregulated effector protein, PstCEP1. This study provides a key resource for understanding the biology and molecular basis of *Pst* responses to wheat HTSP resistance, and PstCEP1 may be used in future studies to understand pathogen-associated molecular pattern-triggered immunity and effector-triggered immunity processes in the *Pst*-wheat interaction system.

## Abstract (precis)

In the present study, we performed transcriptomic analysis to identify differentially expressed genes and effector proteins of Puccinia striiformis f. sp. *tritici* (*Pst*) in response to the high-temperature seedling-plant (HTSP) resistance in wheat. Experimental validation confirmed the function of the highest upregulated effector protein, PstCEP1. This study provides a key resource for understanding the biology and molecular basis of *Pst* responses to wheat HTSP resistance, and PstCEP1 may be used in future studies to understand pathogen-associated molecular pattern-triggered immunity and effector-triggered immunity processes in the *Pst*-wheat interaction system.

## INTRODUCTION

Stripe rust, caused by Puccinia striiformis f. sp. *tritici* (*Pst*), is one of the most devastating diseases in wheat worldwide (1–3). Although fungicides can control the disease effectively, breeding wheat cultivars with durable resistance is a long-term effective method for managing the disease (4). However, adopting cultivars with specific resistance (*R*) genes leads to coevolution in *Pst*, resulting in emergence and rapid build-up of new virulence factors in predominant races, e.g., virulence factors against resistance genes *Yr2*, *Yr9*, *Yr17*, and *Yr27* (3). For instance, new and more aggressive *Pst* races have recently emerged and rapidly established; in addition, these new races appear to better adapt to high temperatures than previous *Pst* races (1). Since 2000, in the eastern United States, new *Pst* isolates were more aggressive than old isolates in terms of important epidemiological characteristics, such as latent period (time from inoculation to the first appearance of spores), lesion size, and spore production on adult plants under both high (12 to 28°C) and low (10 to 18°C) temperatures (5, 6). Temperature can also affect host plant defense, depending on plant development stages. For example, high-temperature adult-plant (HTAP) resistance has been used as a non-race-specific and durable resistance to manage *Pst* on wheat in the United States since the early 1960s (7).

Puccinia striiformis f. sp. *tritici* is an obligate biotrophic pathogen, requiring living plants to complete its life cycle (8). The length of latent period and urediniospore production have been regarded as the most important parameters for quantifying pathogen aggressiveness and horizontal resistance in the wheat-*Pst* pathosystem (9, 10). A full-length cDNA library of those expressed genes in the urediniospores of a particular U.S. *Pst* race was constructed (11). From a total of 42,240 clones, 196 randomly selected genes were sequenced and characterized into 15 functional categories. Similarly, Zhang et al. (12) constructed a cDNA library from germinated urediniospores of a Chinese *Pst* race and characterized 1,118 genes (from 4,798 expressed sequence tags [ESTs]) into eight functional groups. There have been several reports of virulence- or infection-related genes derived from ESTs, mainly from urediniospores, *Pst*-infected tissues, and *Pst* haustoria (11–16). However, the exact molecular mechanisms underlying *Pst* infection and development within host tissues under stress conditions remain unclear.

The interaction between *Pst* and wheat is operated via effector proteins secreted by the pathogens and its targeted proteins in hosts (17). Rust transferred proteins (RTPs) possess protease inhibitor function, and RTP1 has recently been shown to be a structural effector involved in the filamentous hypha formation in the extrahaustorial matrix (18, 19). RTPs were first described in Uromyces fabae (the bean rust pathogen) and shown to translocate from the haustorium to the plant cytoplasm (20). For stripe rust, five candidate effectors were identified in four *Pst* isolates from the United Kingdom and the United States (21). In addition, PEC6, Pst_8713, PstGSRE1, and PSTha5a23 from *Pst* were demonstrated to impair plant immunity (22–25). Nine candidate effectors from *Pst* and Puccinia graminis f. sp. *tritici* (*Pgt*), the wheat stem pathogen, suppressed cell death caused by different elicitors (26). Most of the characterized plant *R* genes belong to those superfamilies encoding proteins with nucleotide binding site/leucine-rich repeats (NBS-LRRs) or receptor-like kinases (RLKs). NBS-LRR and RLK genes usually confer hosts with complete resistance against various pathogens/pests, including biotrophic fungal pathogens (27, 28). Although the classic gene-for-gene interaction has been demonstrated in model plant species, thus far, only four secreted effectors of *Pst* have been confirmed to interact with host targets (22, 29–31).

High-temperature resistance in wheat to *Pst* was first demonstrated in 1985 (32). It can be divided into two types, high-temperature seedling-plant (HTSP) resistance and HTAP resistance (7). Our previous research screened 28 wheat cultivars (lines) with the HTSP resistance from 495 cultivars (lines) (33). Among them, Xiaoyan6 (XY6) is a cultivar with typical HTSP resistance and maintains the resistance over 40 years (34). However, the molecular mechanism of the interaction between *Pst* and the wheat cultivar with HTSP resistance is unclear. In the present study, in order to answer the question why the HTSP resistance in XY6 can be maintained during the long term, we conducted transcriptome sequencing (RNA-seq) and analyzed *Pst* differentially expressed genes (DEGs) during the pathogen incubation period in relation to the HTSP resistance in XY6. In total, 25 DEGs and 34 secreted proteins were identified; one of these proteins (*Pst* candidate effector protein 1, PstCEP1) was validated for its function as a candidate effector. The transcript profile of *PstCEP1* was analyzed under different temperature treatments. *PstCEP1* functions were identified with the bacterial type three secretion system (TTSS)-mediated overexpression and barley stripe mosaic virus (BSMV)-mediated host-induced gene silencing (HIGS). The results indicate that PstCEP1 is a candidate effector and responds to the HTSP resistance in XY6.

## RESULTS

### Overview of the *Pst* transcriptome.

**FIG 1:**
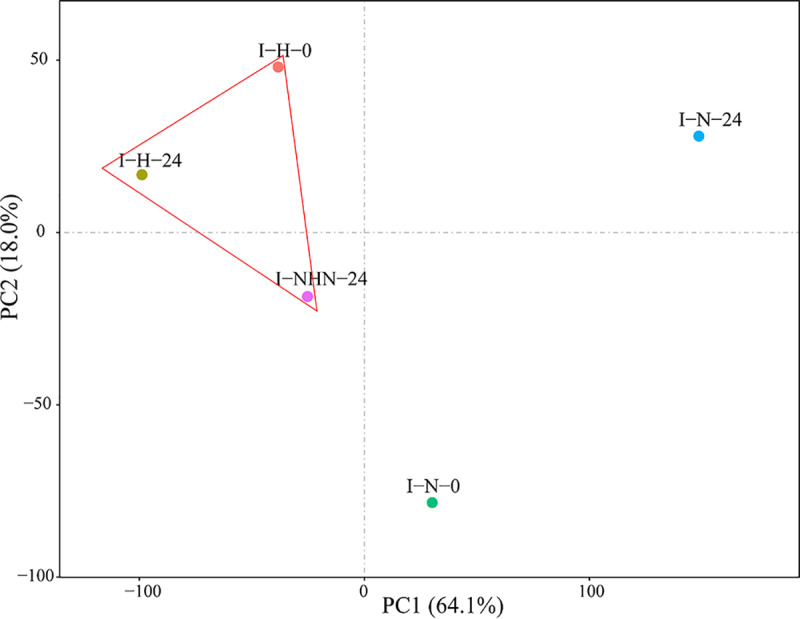
Principal-component analysis (PCA) of the transcriptomes during development of differentially expressed genes (DEGs) of Puccinia striiformis f. sp. *tritici* in response to the high-temperature seedling plant resistance in wheat cultivar XY6. For the principal components 1 and 2, eigenvalues are 64.1% and 18.0%, respectively. The analysis used the expression data from 13,526 genes. Colors indicate different temperature treatments.

The *Pst* transcriptome was profiled and analyzed at two time points (0 and 24 h) on wheat plants inoculated with *Pst*, which were subjected to three treatments: (i) normal temperature (N), (ii) normal-high-normal temperature (NHN), and (iii) high temperature (H). A total of 15 samples, each treatment with three biological replicates, were analyzed with the same sample used for both NHN and N at 0 h. First, we compared the mixture of wheat and stripe rust (CYR32) samples with Chinese Spring wheat genome and then obtained the host genes for the HTSP resistance. Second, we obtained the genes of CYR32 involved in the HTSP resistance by the reads compared with the *Pst-78* genome. We have also calculated the proportion of mapping of host and pathogen according to their genome, respectively. We obtained a total of 340,757,732 reads with an average of 22,717,182 reads per sample. The average mapping rate to Chinese Spring genome was 64.51% (see Table S1 in the supplemental material). In addition, 13,526 genes were predicted when the reads were mapped onto the *Pst*-*78* genome with an average mapping rate of 80.27% (Table S1; Fig. S1). Gene expression levels were expressed as fragments per kilobase of gene per million mapped fragments (FPKM). For nearly a quarter of the genes, the expression levels were in the range of 10 and 100 (FPKM) for each treatment. About 15.21% of genes had high expression levels with FPKM > 100 for the inoculated plants under the N treatment at 24 h (I-N-24), compared with the corresponding value of 5.82% for the NHN treatment (Table 1). The first two principal components (PCs) explained 64.1% and 18.0% of the total variation, respectively. The I-N-0 and I-N-24 samples differed from those high-temperature-inoculated samples (I-H-24 and I-NHN-24) (Fig. 1), indicating that the temperature treatments significantly affected *Pst* gene expression.

**TABLE 1:** Summary of FPKM reads under different treatments^*a*^

Sample	FPKM > 0	0 < FPKM ≤ 1	1 < FPKM ≤ 10	10 < FPKM ≤ 100	FPKM > 100
I-H-0	12,415 (61.28)	2,630 (12.98)	3,184 (15.72)	5,620 (27.74)	981 (4.84)
I-H-24	12,064 (59.55)	3,145 (15.52)	5,164 (25.49)	3,423 (16.90)	332 (1.64)
I-N-0	11,617 (57.34)	1,932 (9.54)	2,467 (12.18)	5,232 (25.82)	1,986 (9.80)
I-N-24	12,194 (60.19)	1,729 (8.53)	2,490 (12.29)	4,894 (24.16)	3,081 (15.21)
I-NHN-24	11,726 (57.88)	2,084 (10.29)	2,909 (14.36)	5,553 (27.41)	1,180 (5.82)

a FPKM, fragments per kilobase of transcript per million mapped fragments. Values represent the number of genes, and values in parentheses are the percentage of genes accounting for all expressed genes in each library.

10.1128/mSphere.00096-20.1 FIG S1 Quality and number of reads in 15 RNA-seq samples from Puccinia striiformis f. sp. *tritici*. Download FIG S1, TIF file, 0.4 MB. Copyright © 2020 Tao et al. 2020 Tao et al. This content is distributed under the terms of the Creative Commons Attribution 4.0 International license.

10.1128/mSphere.00096-20.9 TABLE S1 Values for reads mapped back to the genome (RMBG) of the 15 cDNA libraries. Download Table S1, DOCX file, 0.01 MB. Copyright © 2020 Tao et al. 2020 Tao et al. This content is distributed under the terms of the Creative Commons Attribution 4.0 International license.

### Identification of DEGs and effector candidates of *Pst* in response to the HTSP resistance in XY6.

We used the EdgeR package as implemented in version 2.0.6 to identify DEGs of *Pst* in response to the HTSP resistance in XY6. The false discovery rate (FDR) < 0.05, the absolute value of log_2_ ratio > ±2, and mean abundance logCPM (log_2_ counts per million) > −2 were used as the threshold to identify DEGs between the NHN and H treatments. Twenty-five DEGs were identified, and their functions were annotated based on the Nr database (Table 2). Compared with the H treatment, 24 DEGs were upregulated under the NHN treatment; these DEGs included secreted proteins (PSTG_13342, 18.68-fold; PSTG_01766, 18.17-fold), heat shock protein (PSTG_03423, 17.70-fold; PSTG_09688, 15.45-fold; PSTG_04312, 8.56-fold), thiazole biosynthetic enzyme (PSTG_08211, 16.45-fold), and NMT1 protein (PSTG_07994, 14.85-fold). Only one cell senescence-related gene (PSTG_16995) was downregulated (19.40-fold) in the NHN treatment. All other DEGs were predicted to encode proteins of unknown functions.

**TABLE 2:** Differentially expressed gene analysis of Puccinia striiformis f. sp. *tritici* in response to high-temperature seedling-plant (HTSP) resistance in wheat cultivar XY6^*a*^

Gene ID	logFC (NHN vs H)	logCPM	*P* value	FDR	gi identifier	Nr annotation
PSTG_16995	−19.4034	13.79808	4.03E−15	5.2784E−11	gi|939610964|	Plant senescence-associated protein (Ophiocordyceps unilateralis)
PSTG_01766	18.17161	7.548479	3.18E−11	0.00000518	gi|909614944|	Secreted protein (Melampsora larici-populina 98AG31)
PSTG_00681	10.74884	5.516581	1.06E−08	6.9573E−05	gi|909615994|	Putative oligopeptide transporter (Puccinia sorghi)
PSTG_03423	17.6954	11.46509	5.73E−08	0.00027843	gi|909613273|	Heat shock protein HSS1 (PST-78)
PSTG_07245	8.356523	19.33291	1.10E−05	0.009576	NA	NA
PSTG_04312	8.561816	10.97374	1.97E−07	0.00064561	gi|909612176|	Heat shock protein 90 (PST-78)
PSTG_13342	18.68212	7.234953	3.51E−07	0.00076421	gi|403176169|	Secreted protein (PST-78)
PSTG_08103	17.04667	7.297945	1.46E−06	0.00273005	gi|909608241|	H^+^-transporting ATPase (PST-78)
PSTG_13593	8.226785	8.394477	1.57E−06	0.00293612	gi|909602578|	Hypothetical protein PSTG_13593 (PST-78)
PSTG_10553	8.888591	7.529557	2.04E−06	0.00329264	NA	NA
PSTG_08211	16.44779	12.48827	2.7E−06	0.00353717	gi|909608160|	Thiazole biosynthetic enzyme (PST-78)
PSTG_14495	8.35918	5.29926	4.69E−06	0.00613864	gi|909601646|	Hypothetical protein PSTG_14495 (PST-78)
PSTG_13186	8.300138	9.073804	5.56E−06	0.00660694	gi|909603032|	Hypothetical protein PSTG_13186 (PST-78)
PSTG_08011	7.976068	8.095922	7.61E−06	0.00830146	gi|909608333|	Hypothetical protein PSTG_08011 (PST-78)
PSTG_09688	15.4483	9.175737	0.000031	0.0218622	gi|909606594|	Heat shock protein 4 (*Pgt* CRL75-36-700-3)
PSTG_04871	7.61206	11.07155	3.55E−05	0.02578936	gi|909611816|	Hypothetical protein PSTG_04871 (PST-78)
PSTG_06732	7.711709	8.89047	3.99E−05	0.02705565	gi|403178988|	Phytoene dehydrogenase (Taphrina deformans PYCC 5710)
PSTG_16708	7.59032	10.40818	4.69E−05	0.02919889	gi|909599259|	SSD1 protein (Rhodotorula glutinis ATCC 204091)
PSTG_10642	7.627368	8.854892	4.91E−05	0.02922614	gi|909605686|	Hypothetical protein PSTG_10642 (PST-78)
PSTG_00836	7.543273	10.40451	5.27E−05	0.02997775	NA	NA
PSTG_04894	7.550594	7.815776	5.78E−05	0.03142952	gi|909611535|	Hypothetical protein PSTG_04894 (PST-78)
PSTG_06964	7.548803	6.614308	6.01E−05	0.03142952	gi|909609600|	Hypothetical protein PSTG_06964 (PST-78)
PSTG_02592	9.718921	5.023833	7.14E−05	0.03590758	gi|909614041|	Hypothetical protein PSTG_02592 (PST-78)
PSTG_08802	7.682798	7.272651	8.77E−05	0.04249523	gi|909607600|	Hypothetical protein PSTG_08802 (PST-78)
PSTG_07994	14.85491	11.27838	0.000121	0.04516943	gi|909608499|	Protein NMT1 (PST-78)

a Data were evaluated by using edgeR. False discovery rate (FDR) < 0.05 and fold change > 2 were used for analysis. NA, not available.

**FIG 2:**
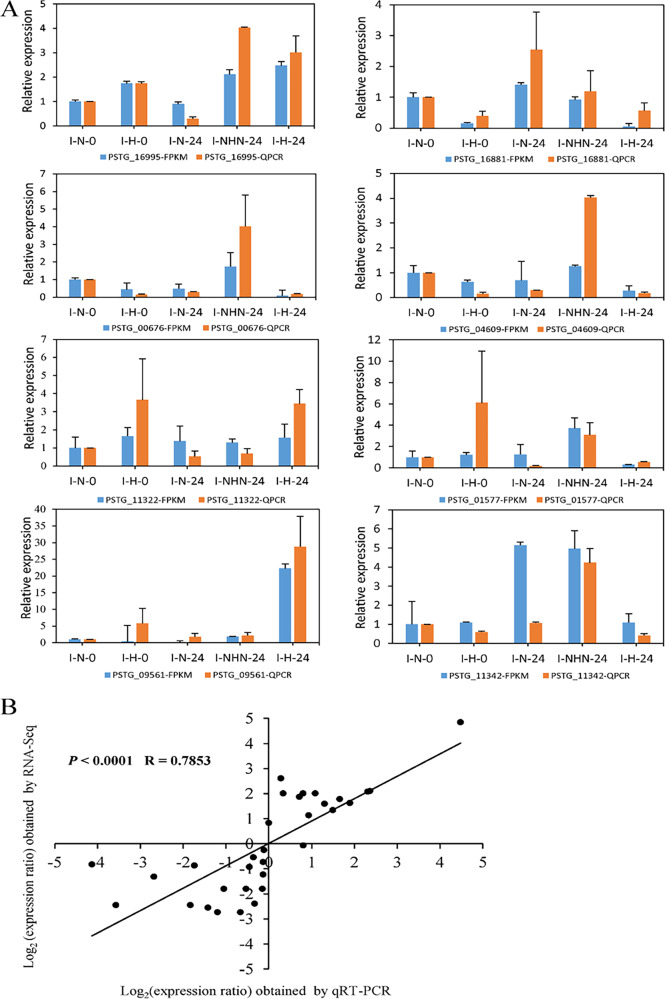
Analysis of transcript level patterns of eight transcripts by qRT-PCR. (A) Relative expression levels of eight randomly selected transcripts through qRT-PCR. The orange histograms represent the relative gene expression levels analyzed using qRT-PCR. The blue histograms represent TMM-FPKM values, which are the relative expression levels of RNA-seq data. The error bars are the mean ± standard error from three replications. (B) Comparison between log_2_ of the differentially expressed gene (DEG) expression ratios obtained from RNA-seq and qRT-PCR.

To verify the gene expression profiles from RNA-seq analysis, eight transcripts were randomly selected from those 25 DEGs for qRT-PCR analysis. The I-N-0 sample was used as a control when calculating relative expression levels. The amplification efficiency and dissolution curve for each gene are given in Fig. S2. The qRT-PCR results were significantly correlated (*P < *0.0001) with the RNA-seq data (Fig. 2A) with a correlation coefficient of 0.79 (Fig. 2B). We searched the 25 DEGs against the KEGG database for classification and functional annotation. These DEGs were mainly involved in immune response, signal transduction, and protein transport pathways (*Q *< 0.05) (Table 3). Similarly, the results of GO enrichment showed that these DEGs were mainly involved in membrane protein, ribonucleotide binding protein, synthesis of nitrogen compounds, and thiamine biosynthesis (Fig. S3, S4, and S5).

**TABLE 3:** KEGG enrichment characterization of DEGs of Puccinia striiformis f. sp. *tritici* in response to the HTSP resistance in wheat cultivar XY6

KEGG class	Pathway	DEGs^*a*^	All genes^*a*^	*P* value	*Q* value	Pathway ID
Immune system	Antigen processing and presentation	3 (42.86)	29 (0.52)	4.39E−06	9.21E−05	ko04612
Metabolism of cofactors and vitamins	Thiamine metabolism	2 (28.57)	13 (0.23)	0.000105	1.10E−03	ko00730
Endocrine system	Estrogen signaling pathway	2 (28.57)	32 (0.57)	0.00066	4.62E−03	ko04915
Metabolism of terpenoids and polyketides	Carotenoid biosynthesis	1 (14.29)	6 (0.11)	0.007524	3.95E−02	ko00906
Environmental adaptation	Plant-pathogen interaction	1 (14.29)	17 (0.31)	0.021192	7.42E−02	ko04626
Immune system	NOD-like receptor signaling pathway	1 (14.29)	17 (0.31)	0.021192	7.42E−02	ko04621
Folding, sorting, and degradation	Protein processing in endoplasmic reticulum	2 (28.57)	218 (3.92)	0.028146	8.44E−02	ko04141
Cancers	Prostate cancer	1 (14.29)	29 (0.52)	0.035919	8.82E−02	ko05215
Infectious diseases	Toxoplasmosis	1 (14.29)	31 (0.56)	0.038355	8.82E−02	ko05145
Infectious diseases	Measles	1 (14.29)	34 (0.61)	0.041999	8.82E−02	ko05162
Infectious diseases	Legionellosis	1 (14.29)	39 (0.7)	0.048046	9.17E−02	ko05134

a Values represent the number of DEGs or all genes, and values in parentheses are the percentage of DEGs or all genes.

10.1128/mSphere.00096-20.2 FIG S2 Amplification efficiency and dissolution curve of each gene. *EF* (black) and *ACT* (blue) were used as reference genes. Red line represents the target gene. Download FIG S2, TIF file, 1.6 MB. Copyright © 2020 Tao et al. 2020 Tao et al. This content is distributed under the terms of the Creative Commons Attribution 4.0 International license.

10.1128/mSphere.00096-20.3 FIG S3 GO enrichment characterization of the differentially expressed genes (DEGs) in Puccinia striiformis f. sp. *tritici* responding to the high-temperature seedling-plant resistance of wheat cultivar XY6. Download FIG S3, TIF file, 0.5 MB. Copyright © 2020 Tao et al. 2020 Tao et al. This content is distributed under the terms of the Creative Commons Attribution 4.0 International license.

10.1128/mSphere.00096-20.4 FIG S4 Functional category of the differentially expressed genes (DEGs) of Puccinia striiformis f. sp. *tritici* responding to high-temperature seedling-plant (HTSP) resistance of wheat cultivar XY6 based on the KEGG database. Download FIG S4, TIF file, 0.7 MB. Copyright © 2020 Tao et al. 2020 Tao et al. This content is distributed under the terms of the Creative Commons Attribution 4.0 International license.

10.1128/mSphere.00096-20.5 FIG S5 Functional classification of the Puccinia striiformis f. sp. *tritici* differentially expressed genes (DEGs) in response to the high-temperature seedling-plant (HTSP) resistance in wheat cultivar XY6 based on the COG database. Download FIG S5, TIF file, 0.5 MB. Copyright © 2020 Tao et al. 2020 Tao et al. This content is distributed under the terms of the Creative Commons Attribution 4.0 International license.

Small secreted proteins have potential roles in the plant-microbe interactions. We analyzed secreted proteins based on the gene expression during the *Pst* infection stage. A total of 1,053 candidate secretory proteins were predicted from 13,526 mapped genes, among which 34 were predicted to have virulence or pathogenicity functions according to the PHI (pathogen-host interaction) database (Table 4). From these 34 genes, we selected the highest-upregulated gene, *PstCEP1* (18.68-fold), as a candidate effector for functional validation.

**TABLE 4:** Secreted protein of Puccinia striiformis f. sp. *tritici* annotated based on the PHI database

Gene ID	Fold change (NHN vs H)	Cys	PHI_MolConn_ID	Gene name	Taxonomy ID	Pathogen name	Phenotype of mutant
PSTG_13342	18.68	4	PHI:5499	Gr-VAP1	31243	*Globodera rostochiensis*	Effector (plant avirulence determinant)
PSTG_02619	−0.58	7	PHI:59	THR1	5462	*Colletotrichum lagenarium*	Reduced virulence
PSTG_07230	2.25	6	PHI:69	CUTA	40559	*Botrytis cinerea*	Unaffected pathogenicity
PSTG_01738	2.57	5	PHI:184	RBT4	5476	Candida albicans	Reduced virulence
PSTG_00510	−1.96	0	PHI:242	CaTPS2	5476	Candida albicans	Reduced virulence
PSTG_08178	−0.51	7	PHI:256	GAS1	318829	Magnaporthe oryzae	Reduced virulence
PSTG_10985	−0.23	4	PHI:278	BCPME1	40559	*Botrytis cinerea*	Mixed
PSTG_13915	−0.13	2	PHI:290	CNB1	5476	Candida albicans	Reduced virulence
PSTG_04011	−10.58	5	PHI:318	SOD1	552467	Cryptococcus gattii	Mixed
PSTG_01920	−0.28	6	PHI:383	SOD5	5476	Candida albicans	Loss of pathogenicity
PSTG_00958	−3.90	6	PHI:407	PBC1	76659	*Pyrenopeziza brassicae*	Loss of pathogenicity
PSTG_11669	−11.88	7	PHI:432	FGL1	5518	Fusarium graminearum	Reduced virulence
PSTG_09464	−0.17	4	PHI:500	YHB1	5476	Candida albicans	Reduced virulence
PSTG_02563	−11.57	3	PHI:548	BcPIC5	40559	*Botrytis cinerea*	Reduced virulence
PSTG_17252	9.52	12	PHI:566	cel2	5017	*Cochliobolus carbonum*	Unaffected pathogenicity
PSTG_16375	−10.90	6	PHI:816	MGG_04582	318829	Magnaporthe oryzae	Reduced virulence
PSTG_00649	−4.57	6	PHI:1087	FGSG_04510	5518	Fusarium graminearum	Unaffected pathogenicity
PSTG_14482	0.10	6	PHI:1228	FGSG_06502	5518	Fusarium graminearum	Lethal
PSTG_10893	−0.24	6	PHI:1466	GzCCAAT006	5518	Fusarium graminearum	Unaffected pathogenicity
PSTG_07350	−12.33	3	PHI:2034	MFP1	318829	Magnaporthe oryzae	Reduced virulence
PSTG_01573	−22.31	4	PHI:2166	CBP1	318829	Magnaporthe oryzae	Unaffected pathogenicity
PSTG_00957	−5.52	6	PHI:2383	MfCUT1	38448	*Monilinia fructicola*	Increased virulence (hypervirulence)
PSTG_11249	0.55	10	PHI:2401	CaRING1	272952	*Hyaloperonospora* *arabidopsidis*	Reduced virulence
PSTG_18873	−0.50	0	PHI:3060	Molrg1	318829	Magnaporthe oryzae	Loss of pathogenicity
PSTG_04848	−2.86	9	PHI:3333	pga7	5476	Candida albicans	Reduced virulence
PSTG_15103	7.97	2	PHI:3608	EPA2	5478	Candida glabrata	Unaffected pathogenicity
PSTG_04949	−2.19	0	PHI:3936	Ss-caf1	5180	Sclerotinia sclerotiorum	Loss of pathogenicity
PSTG_06026	−12.79	5	PHI:3972	CDA	474922	*Colletotrichum* *gloeosporioides*	Unaffected pathogenicity
PSTG_08748	2.19	4	PHI:4010	Plp	55601	Vibrio anguillarum	Unaffected pathogenicity
PSTG_07358	−0.97	5	PHI:4689	pgdA	1639	Listeria monocytogenes	Reduced virulence
PSTG_09484	−0.28	6	PHI:4980	XEG1	67593	*Phytophthora sojae*	Effector (plant avirulence determinant)
PSTG_16871	0.04	6	PHI:4988	Ppt1	5016	*Cochliobolus heterostrophus*	Reduced virulence
PSTG_05144	−0.80	6	PHI:5058	pchB	287	Pseudomonas aeruginosa	Unaffected pathogenicity
PSTG_05115	−5.49	3	PHI:17	ACP	5482	Candida tropicalis	Reduced virulence

### Cloning and sequence characterization of *PstCEP1*.

A 1,321-bp cDNA fragment was isolated from *Pst* via reverse transcription-PCR (RT-PCR) and rapid amplification of cDNA ends (RACE). This cDNA fragment encodes a small protein of 243 amino acids, containing four Cys. The first 25 amino acids at the N terminus of PstCEP1 are predicted to be a signal peptide based on the detection with SignalP ver. 5.0 software (35). The yeast signal sequence trap system (36) was used to confirm the secretory function of this signal peptide. Yeast mutant strain YTK12 cannot grow on the complete minimal plates lacking tryptophan (CMD-W), nor can it grow on the yeast extract, peptone, raffinose, and antimycin A (YPRAA) medium. When the pSUC2T7M13ORI (pSUC2) (36) empty vector was transferred to the YTK12 strain, the tryptophan and nonsecretory sucrase could be encoded, and therefore YTK12 could grow on the CMD-W medium but not on the YPRAA medium. When the pSUC2 vector was linked to a signal peptide that possesses a secretory function, the sucrase could be activated and secreted into the YPRAA medium to hydrolyze the raffinose into glucose, required by strain YTK12. The predicted signal peptide of Phytophthora sojae effector Avr1b (37) was used as a positive control. The results showed that the putative signal peptide of PstCEP1 enabled YTK12 to grow on both the CMD-W and YPRAA media, indicating that PstCEP1 is a secretory protein (Fig. S6).

10.1128/mSphere.00096-20.6 FIG S6 Secretory function verification of the predicted signal peptide of PstCEP1. (A) Features of PstCEP1. SP, signal peptide. (B) The sequence of the signal peptide of PstCEP1 was transferred to the pSUC2 vector and then transformed into yeast strain YTK12. The YTK12 strain without any treatment and empty pSUC2 vector were used as negative controls, and the oomycete effector Avr1b from *P. sojae* was used as a positive control. Only yeast strains capable of secreting invertase can grow on YPRAA medium. Download FIG S6, TIF file, 0.4 MB. Copyright © 2020 Tao et al. 2020 Tao et al. This content is distributed under the terms of the Creative Commons Attribution 4.0 International license.

### Transcriptional expression analysis of *PstCEP1*.

**FIG 3:**
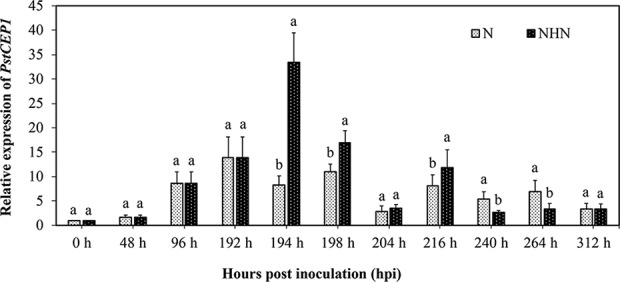
Transcriptional expression analysis of *PstCEP1* during Puccinia striiformis f. sp. *tritici* (*Pst*) infection of wheat by qRT-PCR. N, normal temperature treatment, constant 15°C. NHN, normal-high-normal temperature treatment, 15°C for the first 192 hpi, then 20°C for 24 h, and back to 15°C. The relative gene quantification was calculated by the comparative *C_T_* method with *Pst* endogenous genes *EF1* and *ACT* as internal references. Values and bars represent means (± standard error) from three independent biological replicates. Duncan’s test was conducted among each treatment at the same time point; the different letters indicate significant differences (*P *< 0.05).

A previous study (38) demonstrated that the HTSP resistance in XY6 was activated by exposure of inoculated plants to 20°C for 24 h. Two treatments were therefore performed to confirm transcriptomic results: (i) inoculated plants from 15°C were transferred to 20°C at 192 h postinoculation (hpi) for 24 h and then returned to 15°C, i.e., the NHN treatment, and (ii) the plants remained at 15°C, i.e., the N treatment. The transcriptional expression level of *PstCEP1* was determined in the leaf samples collected at 0 (prior to inoculation), 48, 96, 192, 194, 198, 204, 216, 240, 264, and 312 hpi with qRT-PCR. The expression level of *PstCEP1* increased gradually with the incubation time before the NHN treatment (0 to 192 hpi). After the NHN treatment, the expression level of *PstCEP1* was greater (*P < *0.05) at 194, 198, and 216 hpi than in the N samples, with a peak at 194 hpi (ca. 33.47-fold increase) (Fig. 3).

### Overexpression of PstCEP1 suppresses PCD caused by BAX/INF1 in Nicotiana benthamiana.

**FIG 4:**
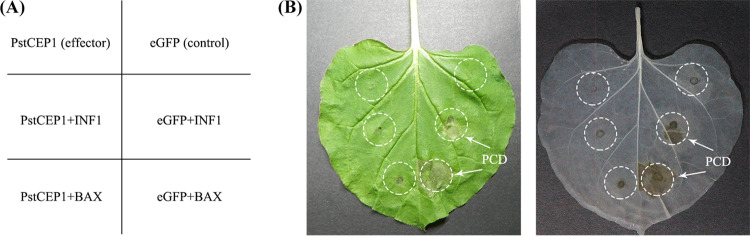
Overexpression of PstCEP1 suppresses programmed cell death (PCD) caused by BAX/INF1 in Nicotiana benthamiana. (A) Diagram of the Agrobacterium tumefaciens infiltration. N. benthamiana leaves were infiltrated with A. tumefaciens cells containing vector PVX carrying eGFP (negative control) or PstCEP1 and inoculated with INF1/BAX 24 h later. (B) Phenotype of the infiltrated leaves. Photos were taken 5 days after infiltration.

BAX is a cell death-promoting protein of the Bcl-2 family in mouse, and INF1 is a pathogen-associated molecular pattern (PAMP) from Phytophthora infestans (39–41). Both proteins can cause programmed cell death (PCD) in N. benthamiana similar to the plant defense-related hypersensitivity reaction (HR). We determined whether overexpressing PstCEP1 in N. benthamiana suppresses PCD caused by BAX/INF1. PstCEP1 and BAX/INF1 were coexpressed in the N. benthamiana leaves, which were assessed 5 days after infiltration (Fig. 4A). Overexpressing PstCEP1 inhibited PCD caused by BAX/INF1, but the control did not (Fig. 4B).

### Subcellular localization of the eGFP-PstCEP1 fusion protein.

**FIG 5:**
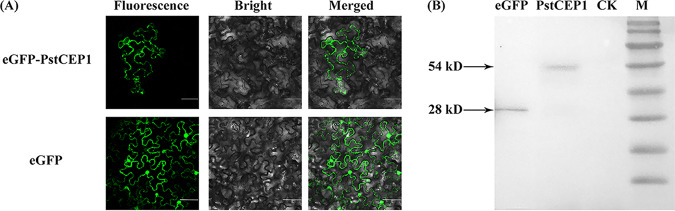
Subcellular localization of PstCEP1 in Nicotiana benthamiana leaves. (A) Constructs of enhanced green fluorescence protein (eGFP) and eGFP-PstCEP1 fusion proteins transformed into Agrobacterium tumefaciens and infiltrated into 6-week-old tobacco leaves. Bars = 50 μm. Fluorescence represents the location of eGFP and eGFP-PstCEP1 fusion proteins, Bright represents the tobacco cells, and Merged represents an overlap between fluorescence and tobacco cells. (B) Western blot analysis of eGFP and eGFP-PstCEP1 fusion protein.

To confirm the subcellular localization of PstCEP1 in host cells, we cloned the open reading frame (ORF) region of *PstCEP1* into the pBINGFP vector. The control (pBINGFP) and pBINGFP-PstCEP1 vectors were then transformed into Agrobacterium tumefaciens strain GV3101 and transiently expressed in N. benthamiana leaves. Fluorescence of enhanced green fluorescent protein (eGFP) was evenly distributed throughout the tobacco cells, and the eGFP-PstCEP1 fusion protein might be located in the cytoplasm (Fig. 5A). The results of Western blotting indicated that both eGFP and eGFP-PstCEP1 fusion protein were successfully expressed in tobacco leaves (Fig. 5B).

### Effect of transient silencing of *PstCEP1* on *Pst* virulence in response to HTSP resistance.

**FIG 6:**
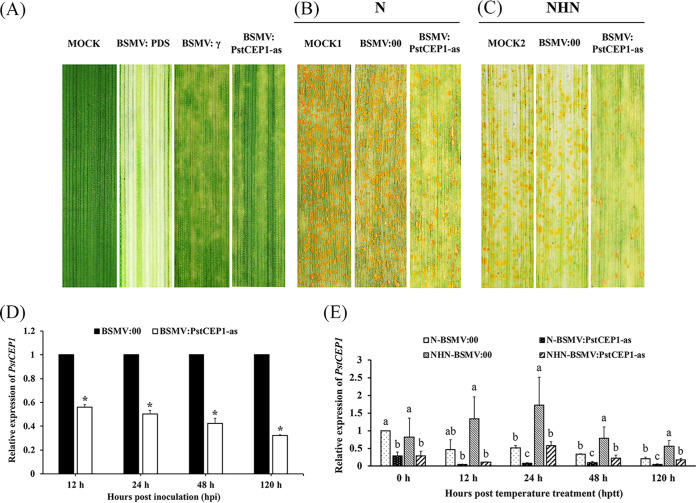
BMSV-mediated host-induced gene silence (HIGS) of *PstCEP1* decreased susceptibility of wheat plants to Puccinia striiformis f. sp. *tritici* (*Pst*). (A) Phenotypes of the fourth leaves of wheat plants after inoculation of FES buffer (MOCK), BSMV:TaPDS, BSMV:γ, and BSMV:PstCEP1-as. Photobleaching of *TaPDS*-silencing leaves and mild chlorotic mosaic symptoms of BSMV-inoculated plants were observed at 11 dpi. (B and C) Phenotypes of the wheat leaves preinoculated with FES buffer (MOCK), BSMV:00 (control), and BSMV:PstCEP1-as, then inoculated with *Pst* race CYR32, and transferred to the N (B) and NHN (C) treatments at 192 hpi. Photos were taken at 336 hpi. (D) Silencing efficiency of *PstCEP1* in the *PstCEP1*-silenced leaves inoculated with CYR32 under N treatment. Leaf samples were collected at 0, 24, 48, and 120 h post-inoculation (hpi) with *Pst*. Values and bars indicate means (± standard error) from three independent biological replicates. Asterisks represent significant differences according to *t* test (*P *< 0.05). (E) Relative transcriptional expression levels of *PstCEP1* in response to the temperature treatments in the *PstCEP1*-silenced and nonsilenced leaves. Leaf samples preinoculated with BSMV were collected at 0, 12, 24, 48, and 120 h post-temperature treatments (hptt).

BSMV-mediated HIGS was performed to knock down *PstCEP1*. The 336-bp specific segment of *PstCEP1* was inserted into BSMV:γ vector and designated as BSMV:PstCEP1-as. All of the BSMV-inoculated plants of XY6 showed stripe mosaic symptoms, and BSMV:PDS-inoculated plants showed bleaching phenotype 12 days after BSMV inoculation (Fig. 6A). Rust lesions were observed on the inoculated leaves of XY6 14 days post-*Pst* inoculation (dpi). The relative expression level of *PstCEP1* indicated that the silencing was successful, and the expression level of *PstCEP1* was suppressed by 43 to 68% by HIGS (Fig. 6D). BSMV:PstCEP1-as reduced sporulation compared with BSMV:00 (control) leaves in both the N and NHN treatments (Fig. 6B and C). The transcriptional expression level of *PstCEP1* was upregulated after the NHN treatment for 12 h compared with those in nonsilenced leaves. Although the NHN treatment induced *PstCEP1* expression in the silenced leaves, the expression level was much lower than in the nonsilenced leaves under the NHN treatment at 24, 48, and 120 h post-temperature treatment (hptt). The results of qRT-PCR were consistent with the observed phenotypes (Fig. 6E).

**FIG 7:**
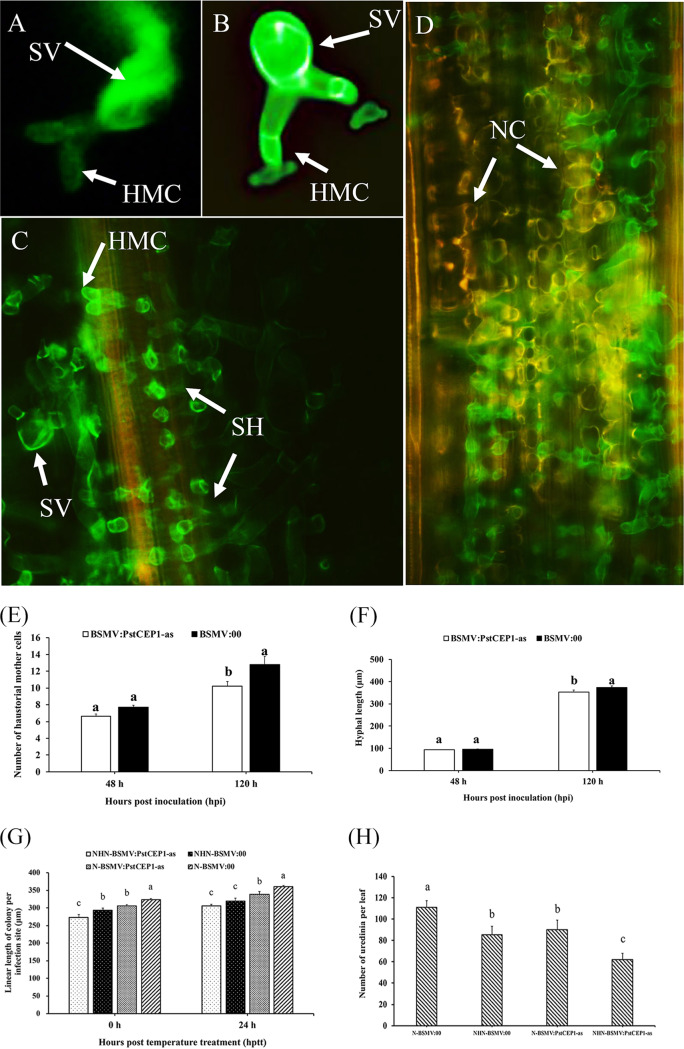
Histological observation of fungal growth in wheat leaves treated with BSMV and inoculated with race CYR32 of Puccinia striiformis f. sp. *tritici*. (A and B) BSMV:γ-inoculated plants and BSMV:PstCEP1-as-inoculated plants at 48 hpi. (C and D) Fungal growth in BSMV:γ-inoculated plants and BSMV:PstCEP1-as-inoculated plants at 120 hpi. SV, substomatal vesicle; HMC, haustorial mother cell; SH, secondary hyphae; NC, necrotic cell. (E and F) Number of haustorial mother cells (E) and hyphal length (F) in wheat leaves treated with BSMV and inoculated with CYR32 at 48 and 120 hpi under N treatment. Values and bars indicate means (± standard error) from three independent biological replicates. The different letters indicate significant differences (*P *< 0.05) according to Duncan’s test. (G and H) Linear length of colony per infection site (G) and the number of necrotic cells in the *PstCEP1*-silenced and nonsilenced leaves (H). Values and bars indicate means (± standard error) from three independent biological replicates with random 30 infection sites per replicate. The different letters indicate significant differences (*P *< 0.05) according to Duncan’s test.

The number of haustorial mother cells and hyphal length were determined at 48 and 120 hpi (Fig. 7A to D). There were no differences at 48 hpi between *PstCEP1*-silenced plants and nonsilenced plants, but the differences (*P < *0.05) were observed at 120 hpi (Fig. 7E and F). In addition, the linear colony length was assessed at 0 and 24 hptt. Colony length in the *PstCEP1*-silenced leaves was shorter (*P < *0.05) for the NHN treatment than for the N treatment, but there were no significant differences between the *PstCEP1*-silenced leaves and nonsilenced leaves (Fig. 7G). There were fewer (*P < *0.05) uredinia in the *PstCEP1*-silenced leaves than in the nonsilenced leaves (Fig. 7H).

### PstCEP1 responds to both the PTI/ETI and HTSP resistance by EtHAn-mediated overexpression in wheat.

To test whether PstCEP1 responds to the host defense-related PAMP-triggered immunity (PTI)/effector-triggered immunity (ETI) and HTSP resistance of the hosts, we overexpressed PstCEP1 in wheat by bacterial TTSS of the Pseudomonas fluorescens effector-to-host (EtHAn) strain (23, 42–44). EtHAn is a nonpathogenic strain of wheat but can cause callose accumulation, which is a characteristic of plant PTI defense response (42). *PstCEP1* was cloned into the effector detect vector 6 (pEDV6), which can release nonbacterial effectors to host cells based on its TTSS function, and then injected into the leaves of wheat cv. Mingxian169 (MX169) mediated by EtHAn (45). MgCl_2_ was used as the blank control, EtHAn and pEDV6-dsRed as the negative control, and pEDV6-AvrRpt2 as the positive control.

**FIG 8:**
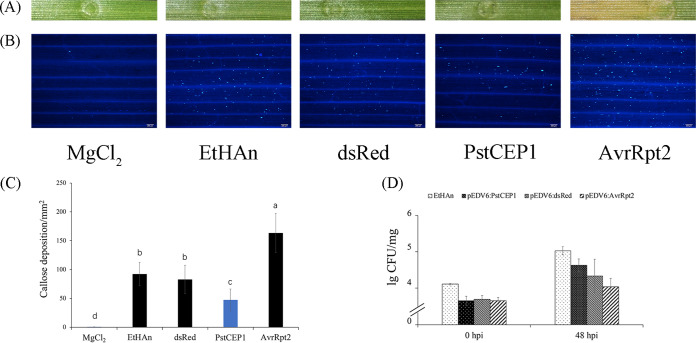
Bacterial type three secretion system (TTSS)-mediated overexpression of PstCEP1 in wheat (MX169) suppressed callose deposition. (A) Phenotypes of wheat leaves infiltrated with MgCl_2_, EtHAn, pEDV6-dsRed, pEDV6-PstCEP1, and pEDV6-AvrRpt2 at 96 dpi. (B) Callose deposition of wheat leaves infiltrated with MgCl_2_, EtHAn, pEDV6-dsRed, pEDV6-PstCEP1, and pEDV6-AvrRpt2 by aniline blue staining. Blue fluorescent spots represent callose deposition. Bars = 200 μm. (C) Average number of callose deposition spots per mm^2^ in wheat leaves inoculated with MgCl_2_, EtHAn, pEDV6-dsRed, pEDV6-PstCEP1, or pEDV6-AvrRpt2. Values and bars indicate means (± standard error) from three independent biological replicates with random 30 mm^2^ per replicate. The different letters indicate significant differences (*P *< 0.05) according to Duncan’s test. (D) Bacterial growth in inoculated wheat leaves. Numbers of bacteria were evaluated at 0 and 48 hpi by plate counting method. Values and bars indicate means (± standard error) from three independent biological replicates.

Inoculation of EtHAn, pEDV6-dsRed, and pEDV6-PstCEP1 did not result in phenotypic changes on the leaves of MX169, but pEDV6-AvrRpt2 did (Fig. 8A). EtHAn, pEDV6-dsRed, pEDV6-AvrRpt2, and pEDV6-PstCEP1 caused callose deposition, but the amount of callose deposition caused by pEDV6-PstCEP1 was smaller (*P < *0.05) than pEDV6-dsRed (Fig. 8B and C). pEDV6-AvrRpt2 could lead to greater callose deposition and cause obvious chlorosis, indicating that PstCEP1 and AvrRpt2 are not the same types of effectors. Furthermore, bacterial counts indicated that EtHAn grew normally in wheat leaves (Fig. 8D).

**FIG 9:**
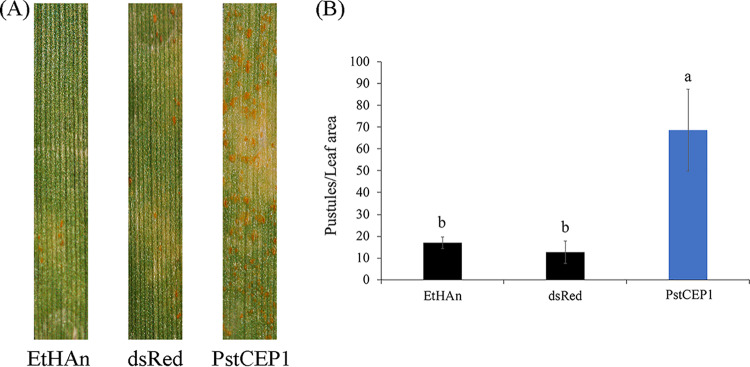
Bacterial type three secretion system (TTSS)-mediated overexpression of PstCEP1 enhanced the virulence of Puccinia striiformis f. sp. *tritici* (*Pst*) in response to high-temperature seedling-plant (HTSP) resistance of wheat cultivar XY6. (A) Disease phenotypes of wheat plants infiltrated with EtHAn wild type, pEDV6-dsRed, and pEDV6-PstCEP1 with *Pst* inoculation at 18 dpi under NHN treatment. (B) Uredinial density in EtHAn wild-type-, pEDV6-dsRed-, and pEDV6-PstCEP1-inoculated wheat plants with *Pst* inoculation at 18 dpi under NHN treatment. Values and bars indicate means (± standard error) from three independent biological replicates. The different letters indicate significant differences (*P *< 0.05) according to Duncan’s test.

Additionally, we overexpressed PstCEP1 in XY6 to study its role in response to the HTSP resistance. The EtHAn-, pEDV6-dsRed-, and pEDV6-PstCEP1-inoculated plants of XY6 were inoculated with *Pst* and incubated at 15°C for the first 192 hpi before being transferred to 20°C for 24 h to activate the HTSP resistance (i.e., the NHN treatment). Samples at 24 hpi, 96 hpi, 216 hpi (24 hptt), and 240 hpi (48 hptt) were taken for histological assessment. Reactive oxygen species (ROS) was produced at 24 hpi, and the differences in ROS accumulation between pEDV6-dsRed and pEDV6-PstCEP1 were not significant. However, pEDV6-PstCEP1 suppressed ROS accumulation at 96 hpi compared with pEDV6-dsRed (*P < *0.05). After the NHN treatment, the ROS of pEDV6-dsRed began to burst, but the ROS accumulation of pEDV6-PstCEP1 remained at the same level as before the NHN treatment. pEDV6-dsRed and pEDV6-PstCEP1 differed (*P < *0.05) in ROS accumulation at 216 and 240 hpi (Fig. S7). In addition, overexpressing PstCEP1 increased the number of uredinia and caused more severe rust development compared with EtHAn and pEDV6-dsRed (*P < *0.05) (Fig. 9). Since the HTSP resistance of XY6 was activated after the NHN treatment, the wheat leaves inoculated with EtHAn and pEDV6-dsRed showed HR and had fewer uredinia compared with inoculation with pEDV6-PstCEP1, indicating that PstCEP1 can respond to HTSP resistance and enhance *Pst* pathogenicity.

10.1128/mSphere.00096-20.7 FIG S7 Bacterial TTSS-mediated overexpression of PstCEP1 in wheat (cv. XY6) suppressed elicitor-trigged immunity (ETI)-associated reactive oxygen species (ROS). (A) H_2_O_2_ accumulation in wheat leaves infiltrated with pEDV6-PstCEP1 and pEDV6-dsRed and inoculated with *Pst* race CYR32 at 96 hpi, 216 (24 h after being transformed to high temperature), and 240 hpi (48 h after being transformed to high temperature), respectively. H_2_O_2_ staining was performed by the 3,3'-diaminobenzidine (DAB) method. Bars = 10 μm. (B) ROS accumulation area per unit area in wheat leaves infiltrated with pEDV6-PstCEP1 and pEDV6-dsRed and inoculated with *Pst* race CYR32 at 24, 96, 216, and 240 hpi. Values and bars represent means (± standard error) from three independent biological replicates with random 30 infection sites per replicate. Asterisks indicate significant differences (*P *< 0.05) between pEDV6-dsRed and pEDV6-PstCEP1 samples according to Duncan’s test. Download FIG S7, TIF file, 0.2 MB. Copyright © 2020 Tao et al. 2020 Tao et al. This content is distributed under the terms of the Creative Commons Attribution 4.0 International license.

## DISCUSSION

Wheat stripe rust occurs frequently in the eastern area of Northwest China where climatic conditions (higher temperature in spring) may have caused the evolution of wheat cultivars containing high-temperature resistance to *Pst*. XY6 is a cultivar with typical HTSP resistance and has maintained this resistance since the 1970s (46). HTSP resistance was expressed in both the seedling-plant and adult-plant periods of wheat growth when seedlings were exposed to 20°C only for 24 h during the initial *Pst* incubation stage (7, 47). In the present study, a total of 25 DEGs of *Pst* in response to the HTSP resistance of XY6 were identified through transcriptomic analysis of the RNA-seq data. KEGG analysis found that the most significant enrichment was the antigen presentation pathway and thiamine metabolism, including several heat shock proteins, N-myristoyl transferase, and thiazole biosynthetic enzyme. DEGs in these pathways may be involved in macromolecular substance synthesis and metabolism in response to the HTSP resistance in XY6 (48, 49).

Recently, *Pst* began to gradually adapt to higher temperatures (5). However, the HTSP resistance in XY6 remains effective. Previous studies suggested that several TaWRKY transcription factors, protein kinases (TaXa21), and resistance proteins (TaRPM1) are involved in the HTSP resistance in XY6 (28, 46, 50, 51). Transcriptomic analysis of the HTSP resistance in XY6 was also performed (38). However, the HTSP resistance is a large and complex network, and its molecular mechanism is still not completely clear. Thus, the most important reason why XY6 maintains the resistance to *Pst* is the complex nature of HTSP resistance, making it difficult for *Pst* to overcome. However, *Pst* attempts to overcome HTSP resistance, and the secretion of effector proteins may be one of the most possible ways.

Effectors play important roles as self-binders, self-modifiers, inhibitors, or activators in the host plants (52). Therefore, we identified 34 secreted proteins of *Pst* according to the transcriptome analysis in the present study. An important candidate effector gene, *PstCEP1*, was found to be upregulated 18.68-fold in response to the host HTSP resistance. We measured the relative expression levels of *PstCEP1* during the infection stages, knocked down *PstCEP1* by BSMV-mediated HIGS, and overexpressed PstCEP1 in wheat by EtHAn-mediated TTSS. The transcriptional expression level of *PstCEP1* was upregulated 2 hptt while the HTSP resistance of XY6 was activating. The HTSP resistance of XY6 was activated after NHN treatment; thus, the wheat leaves inoculated with EtHAn and pEDV6-dsRed showed HR and a small number of uredinia but resulted in a large number of uredinia by pEDV6-PstCEP1, indicating that PstCEP1 can respond to HTSP resistance and enhance the pathogenicity of *Pst*. These results show that *PstCEP1* is highly expressed and involved in the host HTSP resistance process, and they provide significant insight into the pathogenesis of *Pst*. In the previous studies, PEC6, Pst_8713, PstGSRE1, and PSTha5a23 have demonstrated suppression of plant PTI and ETI as a type of effector proteins of *Pst* (22–25). Sixty-nine of 91 haustorial secreted proteins of *Pst* have been proved to suppress PCD in tobacco (53). Moreover, it has been proved that effectors of bacteria, oomycetes, and fungi can suppress PTI-associated PCD and callose deposition, as well as ETI-associated ROS accumulation (22, 26, 54, 55). Our results indicate that PstCEP1 shares a similar function with these effector proteins. PstCEP1 can respond to the HTSP resistance in XY6 and enhance the pathogenesis of *Pst*. However, the HTSP resistance in XY6 is non-race specific and durable (28, 38). XY6 still maintained some level of resistance after overexpressing PstCEP1 compared with the N treatment. The results suggest that the molecular mechanism of the HTSP resistance in XY6 is complicated and not likely to be controlled by a major *R* gene.

HTAP resistance to *Pst* in wheat has been successfully used in the United States for many years (7). Several *Yr* genes have been characterized as conferring HTAP resistance, such as *Yr18*, *Yr29*, *Yr36*, *Yr39*, *Yr48*, *Yr52*, *Yr59*, *Yr62*, *Yr78*, and *Yr79* and several quantitative trait loci (QTL) (56–67). However, most of these resistance genes have not been verified as effective or widely used in breeding in China. Another type of resistance is called all-stage resistance, which is usually qualitative and controlled by a single *R* gene (68). Thus, all-stage resistance in wheat will be overcome in the process of coevolution with *Pst*. In contrast, HTSP and HTAP resistances are non-race specific and durable. HTAP resistance has been demonstrated to be a quantitative trait, which is often controlled by multiple genes (68). Similarly, our results suggest that the phenotype of the HTSP resistance in XY6 is more like a quantitative trait, because a single effector provides only partial virulence against the HTSP resistance in XY6. In addition, conditions for activating the HTSP resistance are exposure to 20°C only for 24 h at the seedling stage, which has the advantage of being easier to identify compared with HTAP resistance. One possible research area in the future is to conduct a cross between XY6 and another cultivar completely susceptible. Breeding wheat cultivars with HTSP resistance is of great importance for control of stripe rust. Our findings provide strong evidence for the understanding of the interaction mechanism between the host HTSP resistance and *Pst* pathogenicity.

### Conclusions.

In the present study, we identified 25 DEGs of *Pst* in response to the HTSP resistance of wheat by RNA-seq. Functional annotation and classification found that these DEGs were related to membrane proteins, mRNA binding proteins, cell membrane transport, and synthesis of cell nitrogen compounds. In addition, we identified 34 secreted proteins, and the highest-expression gene, *PstCEP1*, was used for functional verification. The results show that PstCEP1 is a candidate effector, which has potential virulence function. Furthermore, PstCEP1 is involved in *Pst* response to the HTSP resistance of XY6. Overexpression of PstCEP1 in XY6 can improve the pathogenicity of *Pst*. However, the single effector provides only partial virulence and the HTSP resistance in XY6 is still effective. Breeding wheat cultivars with HTSP resistance is of great importance for control of stripe rust. The present study improves our understanding of the molecular mechanisms of the *Pst*-wheat system.

## MATERIALS AND METHODS

### Plant materials, fungus, bacterial strains, and sample collection.

In the present study, wheat cv. Mingxian169 (MX169) with susceptibility and wheat cv. Xiaoyan6 (XY6) with HTSP resistance again *Pst* (33) were provided by the Institute of Plant Pathology, Northwest A&F University, Yangling, Shaanxi, China. Wheat seeds were provided by Chunlian Li from the College of Agronomy, Northwest A&F University, Yangling, Shaanxi, China. Tobacco variety N. benthamiana seeds were bought from Huayueyang Company (Beijing, China) and self-propagated in the lab. The *Pst* race Chinese Yellow Rust Race 32 (CYR32) was identified and provided by the Institute of Plant Protection, Gansu Academy of Agricultural Science. The EtHAn strain was identified and provided by the College of Plant Protection, Northwest A&F University, Yangling, Shaanxi, China. Propagation and preservation of the materials were done in our lab. Tobacco plants were grown in crocks (10 × 10 × 10 cm^3^) under 16-h light/8-h dark at 25°C. Wheat plants were also grown in crocks (10 × 10 × 10 cm^3^) with planting distance 1.5 cm under rust-free conditions. For infection assay, urediniospores of CYR32 were suspended to a ratio of ∼1:6 to 9 (vol/vol) in sterile distilled water. Two-leaf stage wheat seedlings were brush inoculated with the urediniospore suspension at the first leaf stage and kept in growth chambers (Percival E-30B; Perry, IA, USA) for 24 h in the dark at 10 ± 1°C with relative humidity of 80% (50). To determine the optimal high temperature to induce wheat resistance to CYR32 (the selected temperatures were 20°C), three types of temperature treatments were used to test for resistance to *Pst*: (i) for normal temperature (N), *Pst*-inoculated wheat plants were incubated at persistent normal temperature (15 ± 1°C); (ii) for normal-high-normal temperature (NHN), *Pst*-inoculated wheat plants were kept at 15 ± 1°C until 8 dpi and then switched to high temperature (20 ± 1°C) for 24 h; and (iii) for high temperature (H), wheat plants were incubated at constant high temperature (20 ± 1°C) after *Pst* inoculation. The beginning of leaf sampling time (0 h) for RNA-seq started when seedlings were moved into high-temperature treatment from normal temperature treatment (8 dpi). Leaves of XY6 inoculated with CYR32 under different temperature treatments were sampled. The samples at 8 dpi (0 hptt) and 9 dpi (24 hptt) under different temperature treatments were used for RNA-seq. All samples were frozen immediately in liquid nitrogen and then stored at −80°C until use.

### RNA-seq analysis.

A total of 15 RNA samples including three biological replicates from the three temperature treatments were isolated using TRIzol reagent (Invitrogen, CA, USA) and then treated with DNase I (Thermo Fisher, MA, USA) at a concentration of 1 U/μg. The quality and concentration of the RNA samples were detected using an Agilent 2100 Bioanalyzer (Agilent, Chandler, AZ, USA). After total RNA was extracted, mRNA was enriched using oligo(dT) beads (Epicentre). Then, the enriched mRNA was fragmented into short fragments using fragmentation buffer and transformed into cDNA with random primers by reverse transcription. Second-strand cDNA was synthesized by DNA polymerase I, RNase H, dNTP, and buffer. The cDNA fragments were purified with QIAquick PCR extraction kit and end repaired, poly(A) was added, and the fragments were ligated to Illumina sequencing adapters. The ligation products were size selected by agarose gel electrophoresis, PCR amplified, and sequenced using the Illumina HiSeq 2000 platform by Macrogen (Seoul, South Korea). Paired-end reads were checked and scored according to the Q30 level standard (average Q30 > 90%). To create a comprehensive transcriptome, data trimming and adapter clipping were performed using Trimmomatic (v 0.33) software (69). Reads obtained from the sequencing machines included raw reads containing adapters or low-quality bases which affected the following assembly and analysis. Thus, to get high-quality clean reads, reads were further filtered according to the following rules: (i) removing the adapters of reads, (ii) removing reads containing more than 10% of unknown nucleotides, and (iii) removing low-quality reads containing more than 50% of the low-quality bases. Then, all reads were assembled by mapping to the *Pst-78* genome. The DEG analyses were evaluated using (v3.2.2) software EdgeR after adjusting the data for batch effects (batch as a factor). In order to find DEGs from *Pst* responding to the HTSP resistance in wheat, the difference of temperature, time, and individual plant needed to be eliminated. X_1_ and X_2_ represent 0- and 24-h samples under N treatment, respectively. X_3_ and X_4_ represent the 0- and 24-h samples under NHN treatment, respectively. X_5_ and X_6_ represent 0- and 24-h samples under H treatment (20°C), respectively. To reduce the effects of individual differences, X_1_ and X_3_ used the same samples. X represents gene expression in each sample. α*_h_* (X_4_ − X_3_) represents the value of the gene expression differences between 24 h after high-temperature treatment. β*_T_*, [(X_5_ + X_6_) − (X_2_ + X_1_)]/2, indicates the value of the gene expression differences between the NHN and H treatments. γ_θ_, [(X_6_ + X_2_) − (X_5_ + X_1_)]/2, represents the value of gene expression differences of individuals under the same time point. Thus, the differences between X_4_ and X_6_ samples are DEGs of *Pst* in response to the HTSP resistance of XY6 (see Fig. S8 in the supplemental material). Probability (*P*) values were adjusted for multiple comparisons using false discovery rate (FDR) α < 0.05 (70). Additionally, DEG sets must have possessed a log_2_ fold change (>2 or <−2) under NHN versus H treatment to be considered.

10.1128/mSphere.00096-20.8 FIG S8 Identification method of differentially expressed genes of Puccinia striiformis f. sp. *tritici* in response to the high-temperature seedling-plant (HTSP) resistance of wheat cultivar XY6. Download FIG S8, TIF file, 0.1 MB. Copyright © 2020 Tao et al. 2020 Tao et al. This content is distributed under the terms of the Creative Commons Attribution 4.0 International license.

### Functional annotation and enrichment analysis.

For function annotation, transcripts were subjected to BLASTx (BLAST v2.2.28, E value < 1E^−5^) analysis against protein databases including Nr, Swiss-Prot, KEGG, and COG. After Nr annotation, the Blast2GO program was used to get GO annotation (71). To investigate the metabolic pathway of annotated transcripts, we aligned the transcripts to the KEGG database. GO terms and KEGG pathways with FDR-corrected *P* values of <0.05 were considered statistically significant.

### Identification of effector candidates.

In order to identify the effector candidates of *Pst* in response to the HTSP resistance in wheat, we extracted all the corresponding protein sequences from the transcripts. The conditions for predicting the effector candidates are as follows. (i) Perl Script was employed to screen candidate proteins with a length of 50 to 400 amino acids; (ii) SignalP v4.1, TargetP v1.1, and TMHMM v2.0 were used to screen candidate secretory proteins with signal peptides and without transmembrane domains, respectively; (iii) the predicted secretory proteins were analyzed by cysteine statistics (number of Cys > 3) and Pathogen-Host Interaction Database (PHI) BLAST (E value < 1e^−5^); (iv) the predicted secretory proteins belonging to DEGs in response to the HTSP resistance in wheat based on RNA-seq were identified as effector candidates.

### Total RNA extraction and qRT-PCR analysis.

Total RNA of wheat leaves inoculated with *Pst* race CYR32 at 0, 48, 96, 192, 194, 198, 204, 216, 240, 264, and 312 hpi was extracted using the SV Total RNA isolation system (Promega, Madison, WI, USA). The RNA concentration and quality were detected using Nanodrop 2000 and electrophoresis. First-strand cDNA was synthesized from 1 μg total RNA using the PrimeScript RT reaction system (TaKaRa, Tokyo, Japan). *PstCEP1* was cloned from the cDNA by TransStart KD Plus DNA polymerase (Transgene, Beijing, China). qRT-PCR was performed by iQ5 (Bio-Rad, Hercules, CA, USA) using UltraSYBR mixture (Cowin, Beijing, China) in a volume of 25 μl consisting of 12.5 μl mixture, 9.5 μl ddH_2_O, 2 μl primers, and 1 μl cDNA. Housekeeping genes *EF1* and *ACT* from *Pst* were used as internal reference genes. Data were collected from three independent biological replicates, each consisting of at least three reactions, and negative controls without templates were detected in case of contamination. The amplification efficiency (80 to 100%) of primers was determined (Fig. S2) by LinReg PCR (72). The expression ratio of each gene was calculated by using the relative expression software tool of REST (v2.0.13) (73).

### Secretory function verification of the signal peptide of PstCEP1.

The signal peptide of PstCEP1 was predicted by SignalP Ver 5.0 and validated using the yeast signal trap system (36). DNA fragments encoding the signal peptide were amplified using specific primers (Table S2) and cloned to pSUC2T7M13ORI (pSUC2) vector by ClonExpress II One Step cloning kit (Vazyme, Nanjing, China) (36). The pSUC2-PstCEP1 vector was transformed into the yeast strain YTK12 and screened on the CMD-W (lacking tryptophan) medium; only the YTK12 strain carrying a pSUC2 vector could grow on the CMD-W medium. Positive colonies were replica plated on the YPRAA (using raffinose as carbohydrate source) medium plates for invertase secretion. YTK12 transformed with pSUC2-Avr1b and the empty pSUC2 vector were used as positive and negative controls, respectively.

10.1128/mSphere.00096-20.10 TABLE S2 Primers used in this study. Download Table S2, DOCX file, 0.02 MB. Copyright © 2020 Tao et al. 2020 Tao et al. This content is distributed under the terms of the Creative Commons Attribution 4.0 International license.

### Identification of PstCEP1 suppression of BAX/INF1-induced programmed cell death based on A. tumefaciens infiltration assays.

The open reading frame (ORF) region of *PstCEP1* was amplified using specific primers (Table S2) and cloned to vector PVX106 using the ClonExpress II One Step cloning kit (Vazyme, Nanjing, China). The recombination plasmids of PVX106-PstCEP1, PVX106-eGFP, BAX, and INF1 were each introduced to A. tumefaciens strain GV3101 by electroporation. The harvested A. tumefaciens cultures containing PstCEP1, eGFP, BAX, or INF1 were collected, washed with 10 mM MgCl_2_ 3 times, resuspended in an infiltration medium (10 mM MgCl_2_) to an OD_600_ of 0.6, and then incubated at 28°C in the dark for 2 h prior to infiltration. The A. tumefaciens suspension carrying PstCEP1 and eGFP was infiltrated into N. benthamiana leaves, and the A. tumefaciens suspension containing BAX/INF1 was infiltrated into the same site 24 h later. Phenotypical observation was performed 5 days after infiltration of BAX/INF1. The leaves were decolorized by heating in absolute ethanol. Each assay consisted of at least three leaves on three independent tobacco plants.

### Subcellular localization of the eGFP-PstCEP1 fusion protein.

To *in planta* express the eGFP-PstCEP1 fusion protein, A. tumefaciens strain GV3101 was used to deliver transgenes into N. benthamiana leaves. The ORF region of *PstCEP1* was cloned into the pBINGFP vector using the ClonExpress II One Step cloning kit (Vazyme, Nanjing, China) via BamHI (New England BioLabs, Hitchin, United Kingdom) digestion (Table S2). The recombination vector pBINGFP-PstCEP1 and the control vector pBINGFP were transformed into A. tumefaciens strain GV3101 (Weidibio, Shanghai, China) and cultured on the LB medium for 1 to 2 days, respectively. A. tumefaciens cells carrying pBINGFP and pBINGFP-PstCEP1 were collected, washed 3 times with 10 mM MgCl_2_, and resuspended in an infiltration medium (10 mM MgCl_2_, 10 mM morpholineethanesulfonic acid [MES], and 200 mM acetosyringone, pH 5.6) to an OD_600_ of 0.4. The suspensions were infiltrated into 8-week-old N. benthamiana leaves. Samples were collected 3 to 4 days after infiltration, and microscopic observation was performed using an FV3000 microscope (Olympus, Tokyo, Japan). The total protein of N. benthamiana leaves was extracted using a plant protein extraction kit (Solarbio, Beijing, China) and stored at −80°C until use. Protein extracts were separated by 10% SDS-PAGE, and anti-GFP antibody (Beyotime, Shanghai, China) was used to detect eGFP.

### BSMV-mediated *PstCEP1* gene silencing.

*PstCEP1* gene silencing was performed by BSMV-mediated HIGS (74). A 336-bp specific cDNA of *PstCEP1* was cloned into a BSMV:γ vector via NotI and PacI (New England BioLabs, Hitchin, United Kingdom) digestion, yielding BSMV:PstCEP1-as (Table S2). The silence of phytoene desaturase (*TaPDS*) gene and BSMV:γ empty vector were used as positive and negative controls, respectively. Capped *in vitro* transcripts were prepared from linearized plasmids BSMV:TaPDS, BSMV:PstCEP1, BSMV:γ, BSMV:α, and BSMV:β using the RiboMAX large-scale RNA production systems (Promega, Madison, WI, USA). BSMV virus vectors (α, β, and γ) or BSMV recombinant virus vectors (α, β, γ-*PDS* and α, β, γ-*PstCEP1*) were inoculated onto the second leaves of wheat seedlings. When the leaves of TaPDS-silencing plants were photobleached and the leaves of BSMV:γ and BSMV:PstCEP1-inoculated plants displayed chlorotic mosaic symptoms, the fourth leaves of wheat were inoculated with *Pst* CYR32, and seedlings were then maintained at 15 ± 1°C. *Pst* development was assessed at 14 dpi. Histological observation of *Pst* development was performed as previously described (28).

### Functional verification of PstCEP1.

EtHAn TTSS-mediated overexpression of PstCEP1 was used to detect whether it responds to the PTI/ETI defense and the HTSP resistance of wheat. The ORF region encoding mature protein without the signal peptide of *PstCEP1* was cloned to pEDV6 vector using Gateway cloning (entry vector pDONR 221; primers are listed in Table S2). The recombination vector pEDV6-PstCEP1, positive-control pEDV6-AvrRpt2, and negative-control pEDV6-dsRed were transformed into the EtHAn strain and cultured on KB medium (2% peptone, 1% glycerol, 0.15% K_2_HPO_4_, 0.15% MgSO_4_, and 2% agar) for 1 to 2 days. The harvested wild-type EtHAn culture and EtHAn cultures carrying pEDV6-PstCEP1, pEDV6-AvrRpt2, and pEDV6-dsRed were collected, washed with 10 mM MgCl_2_ for 3 times, and resuspended in an infiltration medium (10 mM MgCl_2_) to an OD_600_ of 0.8. The wild-type EtHAn culture and resuspended EtHAn cultures carrying pEDV6-PstCEP1, pEDV6-AvrRpt2, and pEDV6-dsRed were infiltrated into the second leaves of wheat cv. MX169 using a syringe with the needle removed. Samples of each treatment were collected at 48 hpi, and phenotypes of wheat leaves were examined at 72 hpi. The callose deposition in leaf samples was observed using fluorescence microscopy after 0.05% aniline blue staining (75). The callose deposition levels were determined by counting the number of fluorescent spots per mm^2^ with 30 random sites, all of which were derived from three biological replicates. Bacterial growth levels in XY6 were measured by cutting 50-mg tissues around the infiltrated point and homogenizing them in 200 ml of the inoculation buffer. The bacterial suspensions were diluted and plated on KB solid medium with 50 mg/ml chloramphenicol and gentamicin.

The wild-type EtHAn culture and EtHAn cultures carrying pEDV6-PstCEP1 or pEDV6-dsRed were infiltrated into the second leaves of XY6 to determine the role of PstCEP1 in response to HTSP resistance. The method of infiltration was the same as previously described, and the wheat plants were inoculated with *Pst* race CYR32 at 24 h after the infiltration of EtHAn. The inoculated wheat plants were maintained in a growth chamber at 15°C, transferred to 20°C at 192 hpi for 24 h, and then returned to 15°C. Samples for histological observation were collected at 24, 96, 216 (24 hptt), and 240 (48 hptt) hpi. Diaminobenzidine (DAB) coloration was used to detect hydrogen peroxide accumulation (76). H_2_O_2_ accumulation per unit area in wheat leaves inoculated with CYR32 at 24, 96, 216, and 240 hpi after infiltration with pEDV6-dsRED or pEDV6-PstCEP1 was determined using a BX-51 microscope (Olympus, Tokyo, Japan). Disease phenotypes were observed, and urediniospore quantification was performed in the EtHAn wild-type, pEDV6-dsRed (control), and pEDV6-PstCEP1 inoculated wheat plants at 14 dpi.

### Data analyses.

Software programs SAS v8.01 (SAS Institute Inc., Cary, NC, USA) and SPSS v25.0 (IBM, Chicago, IL, USA) were used to analyze the experimental data. Duncan’s test at *P = *0.05 was used for determining the differences in the related expression of *PstCEP1* as well as histological statistics between treatments at each temporal point.

### Data availability.

The raw data used in the present study for transcriptome assembly and gene expression analysis have been submitted to the NCBI Sequence Read Archive (SRA) database under accession numbers SRR5580869, SRR5580870, SRR5580871, SRR5580872, SRR5580873, SRR5580874, SRR5580875, SRR5580876, SRR5580877, SRR5580878, SRR5580881, SRR5580882, SRR5580883, SRR5580884, and SRR5580886. The sequence of *PstCEP1* has been submitted to GenBank under accession no. MN431201.
