# Supplementary material for: Revealing Differentially Expressed Genes and Identifying Effector Proteins of Puccinia striiformis f. sp. tritici in Response to High-Temperature Seedling Plant Resistance of Wheat Based on Transcriptome Sequencing
Source: mSphere. 2020 Jun 24;5(3):e00096-20. doi: 10.1128/mSphere.00096-20 (PMC7316484; doi:10.1128/mSphere.00096-20)
Supplement: TABLE S1 [file mSphere.00096-20-st001.docx]

**Table S1.** The reads mapped back to genome (RMBG) values of the 15 cDNA libraries

| Samples | Wheat cultivar Xiaoyan6 mapped to Chinese Spring wheat genome | | | |  | Chinese Yellow Rust Race 32 mapped to *Pst*-78 genome | | | |
| --- | --- | --- | --- | --- | --- | --- | --- | --- | --- |
|  | Total reads number | ACE1 | ACE > 1 | OAR |  | Unmapped reads | ACE1 | ACE > 1 | OAR |
| I-H-0-1 | 25167263 | 10.52% | 61.43% | 71.95%  61.26%  62.31%  55.13%  57.26%  61.65%  77.10%  75.88%  62.35%  64.30%  56.72%  81.93%  61.98%  56.85%  60.95% | | 7059417 | 4.69% | 73.06% | 77.75% |
| I-H-0-6-1_1 | 24452234 | 8.48% | 52.78% |  |  | 9472795 | 5.31% | 80.08% | 85.39% |
| I-H-0-6-2_1 | 19511903 | 9.02% | 53.29% |  |  | 7354036 | 4.75% | 66.56% | 71.31% |
| I-H-24-1 | 18390752 | 11.10% | 44.03% |  |  | 8251930 | 4.95% | 67.43% | 72.38% |
| I-H-24-2_1 | 27490451 | 11.18% | 46.08% |  |  | 11749419 | 6.78% | 77.50% | 84.28% |
| I-H-24-6_1 | 22859773 | 10.07% | 51.58% |  |  | 8766723 | 5.09% | 76.96% | 82.05% |
| I-N-0-3 | 20075674 | 3.96% | 73.14% |  |  | 4597329 | 3.17% | 87.86% | 91.03% |
| I-N-0-5_1 | 20123805 | 10.17% | 65.71% |  |  | 4853862 | 3.61% | 69.06% | 72.67% |
| I-N-0-6_1 | 26077533 | 8.10% | 54.25% |  |  | 9818191 | 5.54% | 68.00% | 73.54% |
| I-N-24-0 | 26081348 | 14.65% | 49.65% |  |  | 9311041 | 5.13% | 82.94% | 88.07% |
| I-N-24-1_1 | 22727327 | 10.33% | 46.39% |  |  | 9836387 | 5.82% | 69.86% | 75.68% |
| I-N-24-2_1 | 19391420 | 17.85% | 64.08% |  |  | 3504030 | 3.09% | 78.66% | 81.75% |
| I-NHN-24-0 | 25328783 | 15.47% | 46.51% |  |  | 9630003 | 5.50% | 80.11% | 85.61% |
| I-NHN-24-1_1 | 19001128 | 12.11% | 44.74% |  |  | 8198987 | 4.81% | 76.45% | 81.26% |
| I-NHN-24-2_1 | 24078338 | 7.42% | 53.53% |  |  | 9402591 | 5.25% | 75.97% | 81.22% |

**Note:** The RMBG values of 15 cDNA libraries were based on three biological replicates. The mapping metrics included reads that aligned concordantly exactly once (ACE1), reads that aligned concordantly >1 time (AC > 1) and the overall alignment rate (OAR).
