# Supplementary material for: Revealing Differentially Expressed Genes and Identifying Effector Proteins of Puccinia striiformis f. sp. tritici in Response to High-Temperature Seedling Plant Resistance of Wheat Based on Transcriptome Sequencing
Source: mSphere. 2020 Jun 24;5(3):e00096-20. doi: 10.1128/mSphere.00096-20 (PMC7316484; doi:10.1128/mSphere.00096-20)
Supplement: TABLE S2 [file mSphere.00096-20-st002.docx]

**Table S2.** Primers used in this study.

| Primer Name | Sequence (5’ – 3’) |
| --- | --- |
| PSTG_16881F | TAGCAGGCGGTGGTGATT |
| PSTG_16881R | GCCTGTACATCTACTTGGGATTG |
| PSTG_00676F | GCTGATCCGAAGACTGGTTT |
| PSTG_00676R | AGTATCGGGAAAAGGGGAAA |
| PSTG_04609F | CCGCTGGAAGTGTTGATGG |
| PSTG_04609R | TCTTGATAGGGTCCGTGCTGT |
| PSTG_09561F | CGGAGCACATACCACAGAG |
| PSTG_09561R | GTACAATTGGGGCCACAT |
| PSTG_01577F | TATCGTCCAAGGGGGAGGTA |
| PSTG_01577R | AAGGGCTTGAACAATTCTGG |
| PSTG_13342F | ATGATTTCTGCGACGGAT |
| PSTG_13342R | TAGAGCGAGCTAGAGATATTTG |
| PSTG_16995F | CGAGGGACTAAAGGATCGA |
| PSTG_16995R | GACATTGTCAGGTGGGGAG |
| PSTG_11322F | ATGATTTCTGCGACGGAT |
| PSTG_11322R | TAGAGCGAGCTAGAGATATTTG |
| ACTB_F | CCGCCTTGGTTCTTGACAATGGTT |
| ACTB_R | ATTCCGACCATCACACCCTGATGA |
| EF1_F | TCGTGTCGAAACCGGTACCATCAA |
| EF1_R | AAACCAACGTTGTCACCTGGCAT |
| RACE_13342F | TCCGACAGCCCAACGACTT |
| RACE_13342R | GATGGCTGGTGTTTGGTTG |
| Pst_13342QC-F | CAAGTGATAGCACAACCTCTG |
| Pst_13342QC-R | GGTGTATAGAATGGGTTATCATAGG |
| pBIN-13342-F | ACCCCCGGGGTCGACGGATCCATGGCCGCTCTCTTTTCTGG |
| pBIN-13342-R | TCTAGTTCATCTAGAGGATCCCTAGCGGCGAGCTGGTGCAC |
| pSUC2-13342-F | GAATTCATGGCCGCTCTCTTTTCTGG |
| pSUC2-13342-R | CTCGAGTGCGTGTAGTAGTTGAAGAG |
| BSMV: γ-13342-F | ATATTAATTAAGATGGCTGGTGTTTGGTTG |
| BSMV: γ-13342-R | TATGCGGCCGCCTGAAAGTTGAGGATTTGTGTG |
| PVX-13342-F | TCAGCACCAGCTAGCATCGATATGGCCGCTCTCTTTTCTGG |
| PVX-13342-R | AACCGTTCATCGGCGGTCGACCTAGCGGCGAGCTGGTGCACC |
| pEDV6-13342-F | ggggacaagtttgtacaaaaaagcaggcttcAGCCCATTTGAATCAATCCACC |
| pEDV6-13342-R | ggggaccactttgtacaagaaagctgggtcCTAGCGGCGAGCTGGTGCACC |

Note: F: Forward primer (5'-3'), R: Reverse primer (5'-3'). QC: The full-length gene amplification.
